# Pharmacokinetics and safety of fosfomycin and flomoxef administered as part of neonatal sepsis treatment (NeoSep1 Part 1)

**DOI:** 10.1128/aac.01126-25

**Published:** 2025-12-29

**Authors:** Adrie Bekker, Navarat Panjasawatwong, Louise F. Hill, Wolfgang Stohr, A. Sarah Walker, Sally Ellis, Angela Dramowski, Andrew Whitelaw, Christina Obiero, James A. Berkley, Alexander Makazi, Sithembiso Velaphi, Reenu Thomas, Petronella Magagula, Ilhaam Abrahams, Firdose L. Nakwa, Mohammed M. Barday, Alison Van Kwawegen, Kamla Pillay, Silke Gastine, Joseph F. Standing, Peter Skoutari, Francesca Schiavone, Mike Sharland, Seamus O’Brien, Julia A. Bielicki, Tim R. Cressey, On behalf of the NeoSep Study Team

**Affiliations:** 1 Department of Paediatrics and Child Health, Stellenbosch University 549430, Cape Town, South Africa; 2 Faculty of Pharmacy, University of Payap 293617, Chiang Mai, Thailand; 3 Centre for Neonatal and Paediatric Infection, Institute for Infection & Immunity, City St George’s, London, United Kingdom; 4 MRC Clinical Trials Unit at UCL 524254, London, United Kingdom; 5 Global Antibiotic Research and Development Partnership (GARDP) 542454, Geneva, Switzerland; 6 Division of Medical Microbiology, Department of Pathology, Stellenbosch University and National Health Laboratory Service, Tygerberg Hospital 728610, Cape Town, South Africa; 7 Clinical Research Department, KEMRI-Wellcome Trust Research Programme 285561 , Kilifi, Kenya; 8 Department & University of Oxford/Nuffield Department of Medicine 6396, Oxford, United Kingdom; 9 Department of Paediatrics, Chris Hani Baragwanath Academic Hospital and School of Clinical Medicine, University of the Witwatersrand 37707 , Johannesburg, South Africa; 10 Infection, Immunity and Inflammation, Institute of Child Health, University College London 11700, London, United Kingdom; 11 Department of Pharmacy, Great Ormond Street Hospital for Children 4956 , London, United Kingdom; 12 Paediatric Infectious Diseases and Paediatric Research Centre, UKBB 30280, Basel, Switzerland; 13 AMS-PHPT Research Collaboration, Faculty of Associated Medical Sciences, Chiang Mai University 375881 , Chiang Mai, Thailand; Providence Portland Medical Center, Portland, Oregon, USA

**Keywords:** Neonatal infection, antimicrobial resistance, pharmacokinetics

## Abstract

Neonatal doses for the off-patent antibiotics fosfomycin and flomoxef, which offer coverage against many extended-spectrum beta-lactamase (ESBL)-producing organisms, are based on limited data. We performed a pharmacokinetic (PK) and safety study of fosfomycin and flomoxef to confirm proposed neonatal dosing before further investigation in a trial (NeoSep1, ISRCTN48721236). Neonates with suspected sepsis, weighing more than 1,000 g, were sequentially enrolled into three antibiotic treatment cohorts: fosfomycin and amikacin (Cohort 1), flomoxef and amikacin (Cohort 2), and flomoxef and fosfomycin (Cohort 3), and followed for 28 days. Plasma samples were taken for PK assessment, with population PK modeling and simulations performed. Sixty-two neonates (48/62 [77%] preterm; 48/62 [77%] ≤7 days postnatal age [PNA]) received at least one dose of study antibiotics. Fosfomycin and flomoxef plasma concentrations were best described by a two-compartment and a one-compartment model, respectively, with postmenstrual age and PNA significantly influencing clearance. The probability of target attainment for fosfomycin was 100% for minimum inhibitory concentrations (MICs) of up to 8 mg/L, and for flomoxef, it was 100% for MICs of up to 0.5 mg/L. Adverse events (AEs) were common in this critically ill cohort. Thirteen (21%) neonates developed 19 trial antibiotic-related AEs (17 with grade ≤2, and 2 of grade 3), none of which required modification or discontinuation of allocated treatment. Seven neonates (11.6%) died. In this predominately preterm population, fosfomycin and flomoxef were safe, with drug exposures similar to published studies supporting the proposed doses for the larger, randomized NeoSep1 trial.

This study is registered with ISRCTN48721236.

## INTRODUCTION

Infections are prevalent during the first month of life and represent one of the leading causes of neonatal mortality worldwide (1). In 2017, an estimated 1.3 million cases of neonatal sepsis were reported globally, with approximately 203,000 sepsis-attributable deaths (2). The burden of neonatal infectious diseases is particularly high in sub-Saharan Africa and Asia, where antimicrobial-resistant (AMR) Gram-negative organisms, such as extended-spectrum-beta-lactamase (ESBL)-producing *Klebsiella pneumoniae* (3, 4), are highly prevalent. This pathogen and AMR profile undermine the effectiveness of WHO-recommended first-line (ampicillin or penicillin plus gentamicin) and second-line (third-generation cephalosporins) empiric treatment for neonatal sepsis in low- and middle-income countries (LMICs) (5, 6), underscoring the urgent need for alternative affordable antibiotic regimens.

Fosfomycin and flomoxef are off-patent intravenous (IV) antibiotics that may be used in combination with each other or with amikacin (7–9) to treat neonatal sepsis in settings with a high prevalence of AMR. Fosfomycin is a phosphonic acid derivative with good bactericidal activity against a wide spectrum of Gram-positive and Gram-negative bacteria (10), with approximately 80–90% of the drug recovered unchanged in urine through glomerular filtration (11, 12). Flomoxef is an oxacephem class β-lactam antibiotic with high levels of activity against both Gram-positive and Gram-negative bacteria (10), and 85% of the drug cleared by the kidney unchanged (13). These antibiotics provide good coverage against common AMR strains, such as ESBL-producing Enterobacteriaceae; however, clinical trials adopting an optimized neonatal dosing regimen are needed to confirm the effectiveness and safety of these novel antibiotic regimens.

To address this knowledge gap, the NeoSep1 trial was designed as an open-label, two-part, randomized controlled trial comparing fosfomycin and flomoxef, in combination with each other or with amikacin, to other currently used antibiotic regimens for the empiric treatment of neonatal sepsis. Part 1, reported here, was a run-in pharmacokinetic (PK) and safety study to confirm the safety and therapeutic exposures of the planned fosfomycin and flomoxef dosing regimens, ahead of the Part 2 randomized controlled trial evaluating the effectiveness of these regimens, which is currently underway.

## RESULTS

**Fig 1:**
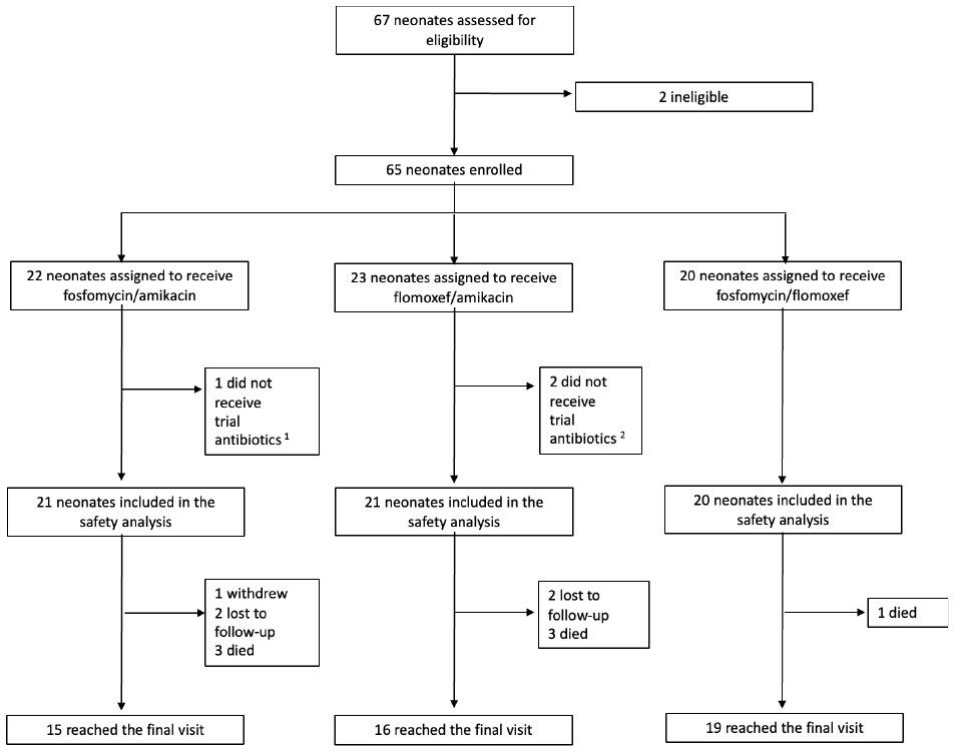
Trial profile of NeoSep1 Part 1. (1) Withdrawn. (2) PK processing was unavailable at the time for two neonates.

Between March and November 2023, 67 neonates were screened for eligibility. Of these, 65 were enrolled in the study (52 from South Africa; 13 from Kenya), with two neonates excluded. Three enrolled neonates were excluded from the analysis as they did not receive any study antibiotics (Fig. 1). The final analysis, therefore, included 62 neonates who received at least one dose of study antibiotics (Table 1), 48 (77%) were preterm, and 48 (77%) were ≤7 days PNA at the time of enrollment.

**TABLE 1:** Baseline characteristics^*a*^

	Cohort 1 Fosfomycin/ amikacin *N* = 21	Cohort 2 Flomoxef/ amikacin *N* = 21	Cohort 3 Fosfomycin/ flomoxef *N* = 20	Total enrolled *N* = 62
Baseline characteristics				
Sex: female	8 (38)	12 (57)	14 (70)	34 (55)
Gestational age at birth (weeks)	31 [27, 38]	34 [26, 40]	32 [30, 42]	32 [26, 42]
Birth weight (grams)	1,285 [875, 3,105]	1,670 [870, 3,310]	1,682 [1,180, 2,970]	1,478 [870, 3,310]
Postnatal age at enrollment (days)	3 [1, 23]	2 [2, 28]	2 [1, 8]	2 [1, 28]
Time in hospital (days)	1 [0, 23]	1 [0, 26]	1 [0, 7]	1 [0, 26]
Baseline chemistry values				
Sodium (mmol/L)	138 [127, 149]	141 [131, 150]	141 [133, 150]	140 [127, 150]
Potassium (mmol/L)	5.2 [3.9, 6.3]	5.0 [4.0, 8.3]	4.8 [3.1, 6.6]	5.0 [3.1, 8.3]
Creatinine (µmol/L)	63 [32, 119]	81 [46, 151]	83 [34, 116]	78 [32, 151]
Total bilirubin (µmol/L)	76 [27, 170]	54 [10, 159]	81 [2, 238]	70 [2, 238]
Non-trial antibiotic treatment for this sepsis episode prior to enrollment				
Ampicillin, gentamicin	9 (43)	9 (43)	16 (80)	34 (55)
Ampicillin, gentamicin, benzylpenicillin			1 (5)	1 (2)
Benzylpenicillin	1 (5)			1 (2)
Benzylpenicillin, gentamicin	1 (5)	7 (33)	1 (5)	9 (15)
Ceftriaxone			1 (5)	1 (2)
Meropenem	2 (10)	1 (5)		3 (5)
Piperacillin/tazobactam, amikacin	8 (38)	4 (19)	1 (5)	13 (21)
Baseline blood culture				
Coagulase-negative staphylococcus^*b*^	1 (5)	1 (5)		2 (3)

^*a*^ Numbers are *n *(%) or median (min, max).

^*b*^ Considered to be contaminants.

At presentation, neonates had a NeoSep severity score ranging between 5 and 9 across all cohorts (Table S1 in supplemental material). All neonates were started on standard-of-care empiric IV antibiotics prior to enrollment, but none for >24 h (eligibility criterion). The type of empiric antibiotic, organisms isolated from blood cultures, and initial chemistry assessments are summarized in Table 1. The median (range) plasma sodium values were 140 (127–150) mmol/L on day 1 and 142 (127–154) on day 5. Laboratory values over time and by cohort are presented in Tables S2 and S3 of the supplemental material. At a median (range) of 7 (1–20) days after enrollment, non-study antibiotics were started in 20 of 62 (32%) neonates for subsequent episodes of sepsis. Table S4 in supplemental material summarizes the reasons for antibiotic initiation, types of antibiotics, and pathogens isolated from sterile-site cultures.

On day 28, 50 of 62 neonates (81%) had completed the study. Seven neonates died during the study period (11%), one neonate (2%) withdrew after receiving a single dose of study antibiotics, and four (6%) were lost to follow-up after hospital discharge and before day 28.

### Pharmacokinetics of fosfomycin and flomoxef

Sixty-one of the 62 neonates enrolled were included in the PK analysis. One neonate was excluded due to difficulties with phlebotomy leading to missing samples. Of the 61 neonates who were included in the PK analyses, 60 had the three pre-specified PK samples collected on day 1, and one neonate only had 2 PK samples taken. Nineteen neonates had a pre-dose sample on day 5.

#### Fosfomycin PK model

**Fig 2:**
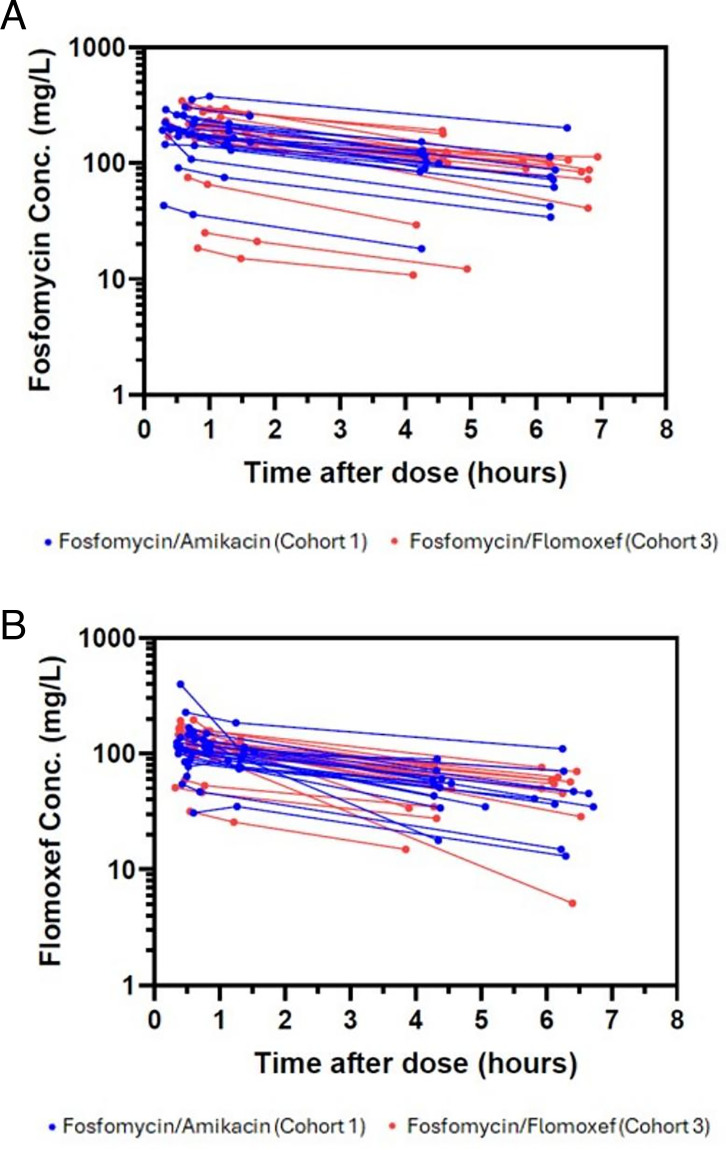
Observed antibiotic concentration versus time. (**A**) Observed fosfomycin concentrations vs time on day 1 for neonates in Cohorts 1 and 3 (*n* = 39). (**B**) Observed flomoxef concentrations vs time on day 1 for neonates in Cohort 2 and 3 (*n* = 40).

One hundred thirty-eight plasma fosfomycin concentrations from 41 neonates (21 in Cohort 1 and 20 in Cohort 3) were available. Two neonates in Cohort 1 had extremely low fosfomycin concentrations on day 1. These were from twins who were dosed on the same day with very low concentrations deemed as outliers and excluded from the analysis. Fosfomycin concentration-time profiles of individual participants are shown in Fig. 2A. The early, middle, and late median concentrations after the first dose were 195, 164, and 98 μg/mL, respectively. A two-compartment model with first-order elimination best described plasma fosfomycin concentrations. Including body weight as an allometric function for both CL and Vd improved the model fit (ΔOFV = −35.62). Renal maturation as a function of PMA was also a significant covariate on CL (ΔOFV = −11.50). Postnatal age (PNA) significantly influenced CL (ΔOFV = −12.66), as did SCR on CL (ΔOFV = −22.70). Although including SCR on CL led to a larger decrease in OFV, based on prior studies, age was more likely to explain the interpatient variability in renal function rather than SCR immediately after birth. Following the inclusion of PNA, the impact of SCR was not significant. The covariates included in the final fosfomycin PK model were body weight, PMA, and PNA. GOF diagnostics of the final model showed adequate fit to the observed data. A few outliers were observed, but no noticeable differences in characteristics/laboratory tests were identified (Fig. S1 in supplemental material). The VPC indicated a good predictive performance (Fig. S2 in supplemental material). Population PK parameter estimates from the final fosfomycin PK model are presented in Table S5 in supplemental material, and the Bayesian post-hoc PK parameters are shown in Table 2. The median plasma fosfomycin AUC_0–24_ was 3,513 mg·h/L on day 1 (*n* = 39) and 2,625 mg·h/L on day 5 (*n* = 16). A boxplot of predicted AUC_0–24_ on days 1 and 5 for fosfomycin is shown in Fig. S5A of supplemental materials.

**TABLE 2:** Fosfomycin and flomoxef post-hoc secondary pharmacokinetic parameters^*a*^

Fosfomycin secondary PK parameters		
	Day 1	Day 5
Number of subjects	39	16
AUC_24 hrs_ (mg·h/L)	3,513.1 (1,215.6–10,008.0)	2,625.0 (1,045.3–5,777.0)
C_max_ (mg/L)	306.8 (15.9–686.1)	359.1 (56.5–782.1)
C_min_ (mg/L)	47.2 (13.2–136.9)	39.8 (10.2–128.3)
T_max_ (minutes)	15.7 (13.0–24.0)	16.1 (15.1–18.9)
Elimination half-life (hours)	6.4 (2.8–78.7)	5.4 (2.5–17.4)

Flomoxef secondary PK parameters		
	Day 1	Day 5
Number of subjects	40	14
AUC_24 hrs_ (mg·h/L)	2,240.3 (752.8–4,578.1)	1,264.4 (765.4–2,234.8)
C_max_ (mg/L)	157.9 (41.6–286.4)	212.8 (142.6–249.2)
C_min_ (mg/L)	30.0 (0.9–69.2)	11.3 (0.5–44.5)
T_max_ (minutes)	19.7 (12.3–40.6)	19.0 (16.2–35.4)
Elimination half-life (hours)	4.1 (1.0–18.3)	1.9 (0.9–4.3)

^*a*^ Data are presented as median (min-max); AUC_0–24_, 4 h area under the concentration-time curve; C_max_, maximum plasma concentration; C_min_, minimum plasma concentration; T_max_, time to maximum plasma concentration.

#### Flomoxef PK model

A total of 134 flomoxef plasma concentrations from 40 neonates (20 in Cohort 2 and 20 in Cohort 3) were included. No flomoxef concentrations were below the LLOQ. The flomoxef plasma concentration-time data are shown in Fig. 2B. The early, middle, and late median concentrations after the first dose were 117, 103, and 50 μg/mL, respectively. Flomoxef plasma concentration data were best described by a one-compartment model with first-order elimination. Inclusion of body weight as an allometric function on CL and Vd improved the model fit (ΔOFV = −15.79), which improved further when including renal maturation as a function of PMA on CL (ΔOFV = −22.91). Incorporating a PNA function on CL led to a larger decrease in the OFV compared to including SCR on CL; however, the parameter estimates were not accurately estimated. Since flomoxef renal drug excretion during the first few days of life is expected to be similar to fosfomycin (i.e., both drugs are primarily excreted through GFR), the ${}_{M}$ (the fraction of CL immediately after birth) and q_N_ (the maturation rate constant for the effect of PNA on CL) parameters were fixed to those from the NeoFosfo trial (0.449 and 0.117 day^−1^, respectively) (14). Following the inclusion of PNA using these parameters, no statistically significant improvement was seen by including SCR on CL. The covariates included in the final flomoxef PK model were body weight, PMA, and PNA. GOF diagnostics showed adequate fit of the final model to the observed data. Several outliers were observed, but no noticeable differences in characteristics/laboratory tests were identified (Fig. S3 in supplemental material). The VPC indicated a good predictive performance (Fig. S4 in supplemental material). The final population PK parameter estimates for flomoxef are shown in Table S6 in supplemental material, and the Bayesian post-hoc PK parameters are shown in Table 2. The median plasma flomoxef AUC_0–24_ was 2,240 mg·h/L on day 1 (*n* = 40) and 1,264 mg·h/L on day 5 (*n* = 14). A boxplot of predicted AUC_0–24_ on days 1 and 5 for flomoxef is shown in Fig. S5B in supplemental materials.

### Model simulations and probability of target attainment for fosfomycin and flomoxef

**Fig 3:**
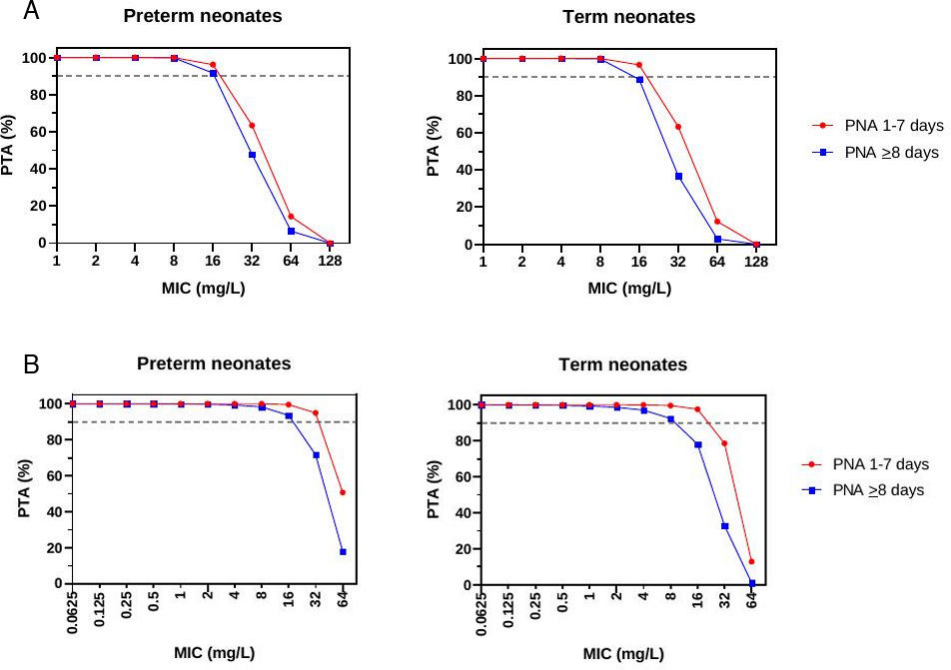
Probability of target attainment (PTA) for different postnatal age (PNA) groups. (**A**) PTA for different PNA groups achieving target AUC_0–24_/MIC ratio of 83, in simulated neonates after administration of fosfomycin, as per dosing schedule. Fosfomycin was given at 100 mg/kg intravenously (IV) twice daily for preterm infants if postnatal age (PNA) was ≤7 days or current weight <1.5 kg, and at 150 mg/kg IV twice daily if PNA was 28 days and ≥1.5 kg. Term neonates received fosfomycin 150 mg/kg IV twice daily. The dashed gray line represents a PTA of 90%. (**B**) PTA for different PNA groups achieving target %fT>MIC of 40 in simulated neonates after administration of flomoxef, as per dosing schedule. Flomoxef was given at 40 mg/kg IV three times daily if PNA ≤7 days, and 50 mg/kg IV three times daily if PNA 28 days, regardless of gestational age. The dashed gray lines represent a PTA of 90%.

The probability of target attainment (PTA) for different PNAs achieving targets of a plasma fosfomycin AUC_0–24_/MIC ratio of 83 (bactericidal efficacy) at specific MICs is shown in Fig. 3A and Table S7 in the supplemental material. For the planned fosfomycin dosage regimen, the PTA is estimated to be 100% for MICs up to 8 mg/L, and approximately 90% when the MIC is 16 mg/L (89% for term neonates with a PNA ≥ 8 days). The PTA of plasma flomoxef %*f*T>MIC ≥ 40% for different PNAs is shown in Fig. 3B and Table S8 in supplemental material. For the planned flomoxef dosage regimen, the PTA is estimated to be 100% for MICs up to 0.5 mg/L and remains over 90% for MICs up to 8 mg/L.

### Adverse events

Forty-eight (77%) of 62 neonates had at least one AE during the study (Table 3). Maximum AE severity was grade 1 or 2 in 28/62 (45%) neonates, grade 3 in eight (13%), and grade 4 in five (8%). Of the seven neonates (11.6%; 95%CI: 5.7–22.8) who died, the median (range) GA was 32 (30–39) weeks, and birth weight was 1,320 (1,120–2,900) g. At the time of death, all were still inpatients, and two were receiving study antibiotics for culture-negative sepsis. No deaths were considered related to study antibiotics (Table S9 in supplemental material).

**TABLE 3:** Adverse events, by study cohort^*a*^

	Cohort 1 Fosfomycin/ amikacin *N* = 21	Cohort 2 Flomoxef/ amikacin *N* = 21	Cohort 3 Fosfomycin/ flomoxef *N* = 20	Total enrolled *N* = 62
Adverse events				
Neonates experiencing at least one adverse event (AE)	16 (76)	14 (67)	18 (90)	48 (77)
Total AEs	77	97	146	320
Neonates experiencing at least one serious adverse event (SAE)	6 (29)	5 (24)	7 (35)	18 (29)
Total SAEs	11	5	7	23
Maximum severity				
Grade 1	2 (10)	3 (14)	1 (5)	6 (10)
Grade 2	7 (33)	6 (29)	9 (45)	22 (35)
Grade 3	3 (14)	0	5 (25)	8 (13)
Grade 4	1 (5)	2 (10)	2 (10)	5 (8)
Grade 5 (death)	3 (14)	3 (14)	1 (5)	7 (11)
Related adverse events				
AE related to fosfomycin	2 (10)		6 (30)	8 (20)
AE related to flomoxef		5 (24)	4 (20)	9 (22)
AE related to amikacin	1 (5)	4 (19)		5 (12)

^*a*^ AE, adverse event; SAE, serious adverse event.

Overall, 23 SAEs were reported in 18 neonates. The most frequent AE (NAESS or non-prespecified events) observed was neonatal sepsis (worsening or new episode), occurring in 26 of 62 (42%) neonates. Among these, 16 of 26 (62%) episodes were blood culture-negative, and 10 of 26 (38%) were blood culture-positive (Table S4 in supplemental material:none occurred while neonates were still receiving study antibiotics). Other commonly reported AEs included hyperbilirubinemia (26/62, 42%), respiratory insufficiency (15/62, 24%), and anemia (14, 23%), consistent with the clinical profile of a predominantly preterm population with clinical sepsis. Study teams reported clinically significant hypernatremia in 9/62 (15%) neonates (twice in two neonates) and hyperkalemia in 10/62 (16%) neonates (twice in one neonate) distributed across all three cohorts.

Thirteen (21%) of 62 neonates experienced a total of 19 AEs related to study antibiotics (Table 3; Table S10 in supplemental material), none of which required modification or discontinuation of the allocated study antibiotics. All were grade 1 or 2, except for 2 grade 3 AEs: hypokalemia and hepatic dysfunction. Three hypernatremia events (all grade one or 2) were related to fosfomycin (*n* = 2) or flomoxef (*n* = 1); two resolved spontaneously, and one decreased to 147 mmol/L 3 days later. One hypokalemia (grade 3) was considered related to fosfomycin, and six renal insufficiency AEs (all grade 1 or 2) were deemed related to either fosfomycin, flomoxef, or amikacin. The fosfomycin-related grade three hypokalemia was observed on day 2 in a 31-week GA (1,320 g) neonate still on fosfomycin with persistent pulmonary hypertension, who died the following day. The second possibly related grade 3 AE was in a 32-week preterm infant who had completed 5 days of fosfomycin and flomoxef. This breastfed infant developed hepatic dysfunction, conjugated hyperbilirubinemia with deranged liver function tests, approximately one month after initiation of study antibiotics (i.e., 3 weeks after stopping study antibiotics). No definitive cause could be established for the abnormal hepatic function with spontaneous resolution one month later.

## DISCUSSION

The NeoSep1 Part 1 study provides the first evidence supporting the safety of fosfomycin and flomoxef used together, or in combination with amikacin, in the empiric treatment of neonatal sepsis within a predominantly preterm population weighing more than 1,000 g. The proposed dosing regimens for fosfomycin and flomoxef achieved adequate exposures consistent with PK parameters in previous neonatal studies. Observed exposures for both agents are anticipated to be sufficient for bacterial killing of isolates with MICs up to 8 mg/L.

Very few neonatal PK studies have been performed to establish optimal dosing of fosfomycin in neonates (15, 16). To improve understanding of fosfomycin use in neonates, the open-label, randomized NeoFosfo trial compared the safety and PK of standard-of-care empirical treatment (ampicillin + gentamicin) for neonatal sepsis versus 100 mg/kg twice daily fosfomycin in 61 neonates (>34 GA weeks and >,1500 g) (14). Fosfomycin plasma drug exposures in the NeoFosfo study were similar to those in our study. However, the NeoFosfo model highlighted potential limitations of the 100 mg/kg twice daily dosing regimen in neonates of older PNA and higher weight. To ensure good PTA for older infants of higher weight, higher fosfomycin doses of 150 mg/kg twice daily were evaluated in those ≥8 days PNA and ≥1.5 kg, as well as in term neonates in our study. The estimated population fosfomycin CL in our study is in agreement with the NeoFosfo study (10.60 vs 8.94 L/h/70 kg, respectively) (14), and with observations in healthy adults (7.20 L/h [17] and 8.70 L/h [18]). Fosfomycin is primarily excreted unchanged via the kidneys. Therefore, nephrogenesis and functional renal maturation during the early postnatal period will influence fosfomycin pharmacokinetics. Indeed, both PMA and PNA significantly impacted fosfomycin CL in this predominantly preterm study population (weight >1,000 g), consistent with previous findings (14).

The flomoxef dose for NeoSep1 Part 1 was based on earlier PK studies performed for licensing (19–23). Our flomoxef exposure was comparable to these earlier studies, but our estimated population CL was 12.6 L/h/70 kg, higher than the 6.25 L/h reported in adults (24). The estimated higher flomoxef CL in our study is likely due to the relatively lower renal function in adults, which corresponds to 67 (54–85) mL/min in one study (24). Similar to fosfomycin, PMA, and PNA significantly affected flomoxef CL due to renal maturation. Given our smaller study, the $q_{M}$ (the fraction of CL on the first day of life) and $q_{N}$ (the post-natal maturation rate constant) were fixed to reported values from the NeoFosfo trial (14).

Susceptibility breakpoints of fosfomycin and flomoxef have not been reported by the Clinical and Laboratory Standards Institute (CLSI) or the European Committee on Antimicrobial Susceptibility Testing (EUCAST), except for a breakpoint of 8 mg/L for IV fosfomycin in urinary tract *E. coli* infections (25, 26). This is due to insufficient clinical and MIC data for establishing clinical breakpoints. In our study, using the proposed dosage regimen for fosfomycin, the PTA was 100% for MICs up to 8 mg/L. Literature has proposed a flomoxef breakpoint of 8 mg/L (10), but outcome data using flomoxef to treat Enterobacterales bacteremia in adults suggest a breakpoint of 1 mg/L may be more appropriate (27). Using the study from this data, we assessed the target attainment of flomoxef with this dosage regimen and found it to be 100% for MICs up to 0.5 mg/L, remaining over 90% for MICs up to 8 mg/L. These findings support the planned dosing for NeoSep1 Part 2. In addition, all three antibiotics in these treatment regimens (fosfomycin, flomoxef, and amikacin) have previously been reported to demonstrate synergistic killing using a hollow fiber infection model (7–9). It is anticipated that these combination antibiotic regimens will increase the target attainment for organisms with higher MICs and could ultimately improve efficacy.

The antibiotic combination regimens tested in NeoSep1 Part 1 were safe and well tolerated in sick term and preterm neonates. Three-quarters of neonates had at least one adverse event (AE), the vast majority of which were assessed as low severity. One-fifth of neonates experienced antibiotic-related adverse events (AEs), none of which required modification or discontinuation of the study antibiotics. Sodium overload has been reported in critically ill adults (28) with the IV fosfomycin formulations containing a high sodium load. At a dose of 100 mg/kg twice daily, IV fosfomycin provides 2.8 mmol/kg/day of sodium, increasing to 4.2 mmol/kg/day at 150 mg/kg twice daily. In comparison, the daily sodium requirement for term neonates ranges from 2 to 3 mmol/kg/day, and for preterm infants, it can be up to 5–7 mmol/kg/day (29). Reassuringly, in the NeoFosfo study, where 100 mg/kg of fosfomycin was administered twice daily to neonates >1,500 g and >34 weeks GA, no reported impact on serum sodium levels was observed (30). In our study, we used a higher dose of 150 mg/kg twice daily in those ≥8 days PNA and ≥1.5 kg, as well as term neonates, based on PK/PD targets from the NeoFosfo study (14). Despite the higher sodium load administered in our study, only two cases of grade 1–2 hypernatremia related to fosfomycin occurred. One hypernatremia event decreased, and one resolved spontaneously.

A strength of our study was that the majority of our cohort consisted of preterm infants within the first week of life, a time when rapid changes in glomerular filtration rate are expected as kidney function matures. Generating plasma concentration data in this preterm population during early life helps to fill knowledge gaps, strengthen the population PK models, and enable more accurate simulations to predict exposure for both fosfomycin and flomoxef for target attainment assessment. Unfortunately, no neonates yielded pathogens from blood culture at baseline, and more data are required to establish susceptibility breakpoints for these antibiotics in neonatal sepsis. Safety profiles were reassuring, despite neonates being critically ill and at moderate to high risk of mortality. The limitation of the population PK analysis in this study was the relatively small number of participants and number of samples on day 5, both of which can contribute to a higher uncertainty in PK parameter estimates. Also, these neonatal PK models cannot be extrapolated to infants and children whose actual body weight, PMA, or PNA on the day of treatment is outside the range of this study.

In summary, in the predominantly preterm population enrolled in NeoSep1 Part 1, fosfomycin and flomoxef exposures shortly after birth were similar to those seen in previously published studies. The exposures for both agents are anticipated to be sufficient for bacterial killing for isolates with MICs up to 8 mg/L, supporting the planned dosing for NeoSep1 Part 2. Part 2 will rank these and other currently used antibiotic combination regimens for empiric treatment of neonatal sepsis in low- and middle-income countries, using 28-day mortality as the primary outcome. Collectively, Part 1 and Part 2 of the NeoSep1 trial aim to generate evidence on effectiveness, safety, cost-effectiveness, and resistance selection, with the potential to inform treatment decisions and support potential inclusion in future WHO empiric guidelines for neonatal sepsis.

## MATERIALS AND METHODS

### Study design

The NeoSep1 Part 1 PK and safety study of fosfomycin and flomoxef recruited neonates sequentially to three antibiotic regimen cohorts: fosfomycin and amikacin (Cohort 1); flomoxef and amikacin (Cohort 2); and flomoxef and fosfomycin (Cohort 3). The enrollment target was 60 neonates with clinical sepsis, 20 per cohort. The study was conducted at three neonatal units: two in South Africa—Tygerberg Hospital (Cape Town) and the Chris Hani Baragwanath Academic Hospital (Johannesburg)—and one in Kenya, Kilifi County Referral Hospital (Kilifi). The NeoSep1 trial (ISRCTN48721236) was approved by the local ethics committees of each site (M22/05/007; 220,509B; KEMRI/SERU/CGMR-C/247/4482) and the relevant national health regulatory bodies. Written informed consent was obtained from the parent(s) or legal guardian(s) of neonates prior to study participation.

### Participant enrollment procedures

Hospitalized neonates (≤28 days) weighing ≥1,000 g, diagnosed with a new episode of clinical sepsis and planned treatment with IV antibiotics, were eligible for enrollment. A NeoSep Severity Score ≥5 (equivalent to moderate-high risk of mortality within 28 days) was required for inclusion in NeoSep Part 1 (Table S11 in supplemental material). This score was derived from a global neonatal sepsis observational study (3) that used baseline neonatal characteristics, supportive care requirements, and clinical signs to identify predictors of mortality at the time of sepsis onset. Neonates were ineligible if routine IV antibiotics had been administered for more than 24 h at the time of planned enrollment. A blood culture, full blood count, urea, electrolytes, C-reactive protein (CRP), creatinine, and liver function tests were collected as part of enrollment if not yet performed as standard of care (Table S12 in supplemental material).

### Dose of fosfomycin, flomoxef, and amikacin

Fosfomycin was supplied as a 40 mg/mL powder for solution containing 14.4 mmol/330 mg sodium per gram. It was administered at 100 mg/kg IV twice daily for preterm infants (<37 weeks gestational age [GA]) if PNA was ≤7 days or current weight was <1.5 kg, and at 150 mg/kg IV twice daily if PNA ≥8 days and ≥1.5 kg. PNA was calculated with day of birth as day 1. Term neonates received fosfomycin 150 mg/kg IV twice daily. This dosing was informed by recent data from the NeoFosfo trial (14). Flomoxef was supplied in 0.5 g/10 mL and 1 g/10 mL powder for solution. The dose of flomoxef, based on the licensed dose, was 40 mg/kg IV three times daily if PNA ≤7 days, and 50 mg/kg IV three times daily if PNA ≥8 days, regardless of GA (31). Amikacin was given at a dose of 15 mg/kg IV once daily, following the WHO AWaRe dosing recommendations for neonatal sepsis (32). If a neonate deteriorated, or failed to respond to the study antibiotics, the local clinician could initiate a second-line antibiotic regimen of their choice.

### Pharmacokinetic sampling

Each neonate had three blood samples drawn for PK sampling on day 1 of study. Participants were randomized to one of eight potential blood sampling schedules using a pre-prepared randomization list: early (5 or 15 min), middle (30 or 60 min), and late (4 or 6 h) blood sampling, following the first dose of study antibiotics. A pre-dose blood sample was drawn on day 5 if the neonate was still receiving study antibiotics (Table S2 in supplemental material). Fosfomycin and flomoxef plasma concentrations were quantified using validated liquid chromatography-tandem mass spectrometry (LC-MS/MS) assays (Analytical Services International [ASI], London, UK). The lower limit of assay quantification (LLOQ) was 5.0 mg/L for fosfomycin and 0.1 mg/L for flomoxef.

### Population pharmacokinetic analysis

PK models for both fosfomycin and flomoxef were developed using nonlinear mixed-effects regression, and plasma concentration-time data were fitted using the first-order conditional estimation method with interaction (NONMEM version 7.5, Icon Development Solution, Ellicott City, MD). Pirana software, a graphical interface for NONMEM, was utilized to run the individual models (V.2.9.9, https://www.certara.com/), and diagnostic plots were generated using R version 4.4.3. Fosfomycin and flomoxef concentration data were converted into natural logarithms for analysis, and various PK structural models were evaluated. The Akaike Information Criterion (AIC) was used to assess non-nested models. Nested models were compared using the minimal value of the objective function value (OFV) provided by NONMEM. An OFV decrease of 3.84 points (i.e., ∆OFV –3.84) corresponds to a statistically significant difference between hierarchical models using a likelihood ratio test (*P*-value ≤0.05, chi-square distribution with one degree of freedom). Inter-individual variability (IIV) was described assuming a log-normal distribution, and an additive model (on a natural logarithmic scale) was applied to describe unexplained residual variability. Covariates anticipated to impact PK parameters were assessed for inclusion in the models: body weight was added using a fixed allometric function on clearance (CL) (exponent fixed to 0.75) and volume of distribution (Vd) (exponent fixed to 1) (33). Since both fosfomycin and flomoxef are primarily excreted unchanged via the kidneys (17), a renal maturation function on CL was tested using an equation that describes the evolution of the glomerular filtration rate from before birth through 1.5 years of life (equation 1) (34). This function, which includes postmenstrual age (PMA), has previously been applied to fosfomycin and meropenem neonatal PK models (14, 35).


(1) $$Maturation=\frac{PMA_{i}^{3.4}}{47.7^{3.4}\;+\;PMA_{i}^{3.4}}$$

Although equation 1 can explain the maturation of renal function in early life, other covariate(s) regardless of GA may also affect the maturation of CL during the first weeks of life. The specific influence of PNA on CL is tested using equation 2 (14, 36), with ${}_{M}$ representing the fraction of CL immediately after birth and ${}_{N}$ the maturation rate constant for the effect of PNA on CL. The impact of serum creatinine (SCR) on CL was assessed (equation 3), where measured SCR was standardized by typical SCR (TSCR, equation 4) (14, 35, 37).


(2) $$PNA_{function}=\theta_{M}+\left(1-\theta_{M}\right)x\left(1-e^{-PNA_{i}x\theta_{N}}\right)$$

(3) $$SCR_{function}={\left(\frac{measured\;SCR}{TSCR}\right)}^{\theta_{SCR}}$$

(4) $$TSCR\;\left(\mu mol/L\right)=-2.37330-\left(12.91367xlog_{e}PNA_{years}\right)+\left(23.93581xPNA_{years}^{0.5}\right)$$

The influence of participant covariates on PK parameters was tested using the standard stepwise forward inclusion and backward elimination model building procedure. Goodness-of-fit (GOF) plots and prediction-corrected visual predictive check (VPC) were used during model-building. Sampling importance resampling (SIR) was used to evaluate parameter uncertainty and model robustness (38).

### Pharmacokinetic model simulations and probability of target attainment (PTA)

Monte Carlo simulations were performed using the final models to predict steady-state fosfomycin and flomoxef exposures, i.e., 24-hour area under the concentration-time curve (AUC_0–24_) and the plasma drug concentrations at specific time points. The characteristics of the neonates (GA, PMA, PNA, and body weight) for the simulation data set were derived from three studies: (i) NeoSep1 Part 1, (ii) NeoFosfo (14), and (iii) NeoOBS (3), generating a total of 2,943 neonates. Simulations (50 × 2,943 = 147,150 neonates) were performed using the fosfomycin and flomoxef doses administered during NeoSep1 Part 1. The pharmacokinetic/pharmacodynamic (PK/PD) targets for fosfomycin were an AUC_0–24_/minimum inhibitory concentration (MIC) ratio of >83 (a 1-log kill dose against Enterobacteriaceae) (39), and for flomoxef, it was the free plasma concentration exceeding the MIC for >40% of the dosing interval (%*f*T>MIC)—previously assessed against ESBL-producing *E. coli* (40). The unbound (free) fraction for flomoxef was fixed to 65% (10). The MIC values for fosfomycin were 1, 2, 4, 8, 16, 32, 64, and 128 mg/L, and for flomoxef, they were 0.06, 0.12, 0.25, 0.5, 1, 2, 4, 8, 16, 32, and 64 mg/L. The AUC_0–24_/MIC for individual neonates was calculated for fosfomycin, and individual %*f*T>MIC was calculated for flomoxef, with relevant PTA calculated for each MIC.

### Pharmacokinetic sample size

The sample size and appropriateness of sample timings were calculated using stochastic simulation and estimation to ensure at least 80% power to estimate CL and Vd with 20% precision. For fosfomycin, the model developed from the NeoFosfo trial was used (14). For flomoxef, a simulation-estimation analysis was carried out, scaling a published adult population PK model (41) to a neonatal reference population by applying weight-based allometry to CL and Vd terms (42), and using previously established maturation functions to further define CL maturation based on PNA and PMA. Prior to the simulation-estimation analysis, the scaled model was inspected against published neonatal flomoxef PK profiles (43) to verify applicability of the scaling.

### Safety assessment and analysis

Clinical and safety evaluations were conducted daily while receiving study antibiotics. Laboratory tests were repeated on day 5 post-enrollment (Table S2 in supplemental material).

AEs were graded using the clinically based International Neonatal Consortium Neonatal Adverse Event Severity Scale (NAESS) (44). This scale defines severity criteria for 35 AEs based on their absence/presence (plus severity grade, if present). Furthermore, AEs not included in NAESS (i.e., not prespecified) were also reported and graded if present, following the NAESS general grading recommendations for “Any other AE.” Laboratory abnormalities were reportable as AEs only when deemed clinically significant by the responsible clinician. Causality assessments were undertaken by trained study personnel against the reference safety information.

AE data were summarized overall and by treatment cohort: number (%) of participants experiencing an AE, number (%) of participants experiencing an adverse reaction, maximum AE grade per participant, and AEs by body system, severity grade, and relationship to study treatment. Categorical data were summarized using the number and percentages, and continuous data were presented using the mean and standard deviation, or median and quartiles and range. Time from enrollment to death was analyzed using Kaplan-Meier methods. All participants were followed through 28 days.

## Data Availability

Data are available upon request to Adrie Bekker (email address: adrie@sun.ac.za) with the agreement of the NeoSep Study Team.

## SUPPLEMENTAL MATERIAL

Supplemental material aac.01126-25-s0001.docx Tables S1 to S12; Fig. S1 to S5.

ASM does not own the copyrights to Supplemental Material that may be linked to, or accessed through, an article. The authors have granted ASM a non-exclusive, world-wide license to publish the Supplemental Material files. Please contact the corresponding author directly for reuse.
