# Supplementary material for: Pharmacokinetics and safety of fosfomycin and flomoxef administered as part of neonatal sepsis treatment (NeoSep1 Part 1)
Source: Antimicrob Agents Chemother. 2025 Dec 29;70(2):e01126-25. doi: 10.1128/aac.01126-25 (PMC12888880; doi:10.1128/aac.01126-25)
Supplement: Supplemental material — Tables S1 to S12; Fig. S1 to S5. [file aac.01126-25-s0001.docx]

# **Supplementary materials**

Supplementary Table 1: NeoSep Severity Score: factors and overall score at presentation

|  | **Cohort 1 fosfomycin/ amikacin**  **N=21** | **Cohort 2 flomoxef/ amikacin**  **N=21** | | **Cohort 3 fosfomycin/ flomoxef**  **N=20** | | **Total**  **N=62** |
| --- | --- | --- | --- | --- | --- | --- |
| Time in hospital (days) | 1 [0, 23] | 1 [0, 26] | | 1 [0, 7] | | 1 [0, 26] |
| Gestational age at birth ^a^ | 31 [27, 38] | 34 [26, 40] | | 32 [30, 42] | | 32 [26, 42] |
| Preterm | 19 (90%) | 14 (67%) | | 15 (75%) | | 48 (77%) |
| Birth weight (g) | 1285 [875, 3105] | 1670 [870, 3310] | | 1682 [1180, 2970] | | 1478 [870, 3310] |
| Congenital anomalies? ^b^ |  |  | |  | |  |
| None | 19 (90%) | 19 (90%) | | 17 (85%) | | 55 (89%) |
| Minor | 2 (10%) | 2 (10%) | | 3 (15%) | | 7 (11%) |
| Temperature (°C) | 36.6 [34.8, 37.2] | 36.5 [34.3, 37.0] | | 36.7 [34.1, 37.7] | | 36.6 [34.1, 37.7] |
| <35.5 | 3 (14%) | 6 (29%) | | 3 (15%) | | 12 (19%) |
| 35.5-37.9 ^c^ | 18 (86%) | 15 (71%) | | 17 (85%) | | 50 (81%) |
| Respiratory support? ^d^ |  |  | |  | |  |
| No | 4 (19%) | 2 (10%) | | 2 (10%) | | 8 (13%) |
| Oxygen supplementation only | 6 (29%) | 11 (52%) | | 6 (30%) | | 23 (37%) |
| CPAP | 10 (48%) | 6 (29%) | | 10 (50%) | | 26 (42%) |
| HFNC | 1 (5%) | 1 (5%) | | 0 (0%) | | 2 (3%) |
| Invasive ventilation | 0 (0%) | 1 (5%) | | 2 (10%) | | 3 (5%) |
| Abdominal distension? |  |  | |  | |  |
| Yes | 6 (29%) | 2 (10%) | | 0 (0%) | | 8 (13%) |
| Difficulty in feeding? |  |  | |  | |  |
| None | 6 (29%) | 4 (19%) | | 2 (10%) | | 12 (19%) |
| Yes, difficulty in feeding or  feeding intolerance | 3 (14%) | 5 (24%) | | 4 (20%) | | 12 (19%) |
| Not fed at all ^e^ | 12 (57%) | 12 (57%) | | 14 (70%) | | 38 (61%) |
| Evidence of shock? |  |  | |  | |  |
| Yes | 0 (0%) | 1 (5%) | | 1 (5%) | | 2 (3%) |
| Lethargy or reduced or no movement? |  |  | |  | |  |
| None | 7 (33%) | 10 (48%) | | 14 (70%) | | 31 (50%) |
| Lethargy only | 1 (5%) | 5 (24%) | | 5 (25%) | | 11 (18%) |
| Movement only on stimulation | 13 (62%) | 6 (29%) | | 1 (5%) | | 20 (32%) |
| NeoSep Severity Score | 6 [5,9] | | 6 [5,8] | 6 [5,8] | 6 [5,9] | |
| NeoSep Severity Score: |  | |  |  |  | |
| 5 | 5 (24%) | | 10 (48%) | 7 (35%) | | 22 (35%) |
| 6 | 7 (33%) | | 5 (24%) | 9 (45%) | | 21 (34%) |
| 7 | 2 (10%) | | 3 (14%) | 2 (10%) | | 7 (11%) |
| 8 | 3 (14%) | | 3 (14%) | 2 (10%) | | 8 (13%) |
| 9 | 4 (19%) | | 0 (0%) | 0 (0%) | | 4 (6%) |

*Note: numbers are N (%) or median [range]. CPAP: continuous positive airway pressure. HFNC: high-flow nasal cannula. ^a^ weeks; ^b^ Major congenital anomalies were an exclusion criterion; ^c^ No neonate had a temperature of 38°C or higher at presentation; ^d^ If a neonate received multiple ways of support, the highest level of support was reported; ^e^ not fed at all orally.*

Supplementary Table 2: Laboratory values at baseline and Day 5 of antibiotics

|  | **N** | **Baseline** | **N** | **On Day 5** |
| --- | --- | --- | --- | --- |
| Sodium (mmol/L) | 56 | 140 (135, 142) [127, 150] | 29 | 142 (139, 146) [127, 154] |
| Potassium (mmol/L) | 54 | 5.0 (4.3, 5.4) [3.1, 8.3] | 27 | 4.5 (3.8, 5.1) [2.8, 6.3] |
| BUN (mmol/L) | 50 | 1.8 (1.3, 2.7) [0.6, 6.4] | 27 | 1.2 (0.9, 2.0) [0.2, 4.7] |
| Creatinine (µmol/L) | 54 | 78 (61, 92) [32, 151] | 29 | 60 (48, 76) [32, 90] |
| CRP (mg/L) | 55 | 1.0 (1.0, 3.0) [0.1, 89.0] | 20 | 1.9 (1.0, 4.1) [0.1, 18.0] |
| ALT (U/L) | 48 | 11 (7, 15) [5, 86] | 13 | 11 (8,19) [7, 35] |
| AST (U/L) | 44 | 64 (38, 77) [24, 319] | 14 | 36 (27,40) [19, 59] |
| Total bilirubin (µmol/L) | 54 | 70 (35, 96) [2, 238] | 30 | 114 (73, 154) [12, 278] |
| Hemoglobin (g/dL) | 60 | 16.2 (14.2, 17.2) [8.5, 21.5] | 18 | 15.2 (12.5, 15.80) [9.3, 17.1] |
| RBC (x10^12^ cells/L) | 60 | 4.3 (3.9, 4.8) [2.6, 6.1] | 18 | 4.1 (3.2, 4.6) [2.8, 5.5] |
| Platelets (x10^9^ cells/L) | 60 | 256 (186, 320) [30, 775] | 18 | 181 (129, 282) [15, 369] |
| WBC (x10^9^ cells/L) | 60 | 11.4 (7.5, 15.3) [3.7, 36.7] | 18 | 6.5 (5.1, 9.3) [2.4, 68.7] |
| Neutrophils (x10^9^ cells/L) | 58 | 5.7 (2.9, 8.1) [0.9, 26.8] | 16 | 2.2 (1.3, 4.0) [0.7, 28.0] |

*Numbers presented are N (%) or median (IQR) [range].*

Supplementary Table 3: Day 5 laboratory values by cohort

|  | **Cohort 1**  **fosfomycin/**  **amikacin** | **Cohort 2**  **flomoxef/**  **amikacin** | **Cohort 3**  **fosfomycin/**  **flomoxef** |
| --- | --- | --- | --- |
| Sodium (mmol/L) | N=7 | N=9 | N=13 |
|  | 139 [127, 147] | 144 [131, 148] | 143 [137, 154] |
| Potassium (mmol/L) | N=6 | N=8 | N=13 |
|  | 4.8 [3.7, 5.2] | 4.6 [3.7, 6.3] | 4.0 [2.8, 5.4] |
| Creatinine (µmol/L) | N=6 | N=9 | N=14 |
|  | 54 [36, 84] | 51 [32, 90] | 64 [37, 87] |
| Total bilirubin (µmol/L) | N=8 | N=9 | N=13 |
|  | 90 [12, 154] | 97 [17, 255] | 129 [20, 278] |

Supplementary Table 4: Neonates who started non-trial IV antibiotics or antifungals post-enrolment

|  | **Cohort 1**  **fosfomycin/**  **amikacin**  **N=6** | **Cohort 2**  **flomoxef/**  **amikacin**  **N=5** | **Cohort 3**  **fosfomycin/**  **flomoxef**  **N=9** | **Total**  **Enrolled**  **N=20** |
| --- | --- | --- | --- | --- |
| **Start of non-trial antibiotics** | | | | |
| Days after enrolment | 9 [4, 20] | 4 [1,7] | 6 [2,10] | 7 [1, 20] |
| **Reason for starting non-trial antibiotic treatment** | | | | |
| Suspected new onset clinical sepsis | 5 (83) | 3 (60) | 5 (56) | 13 (65) |
| Not responding to empiric trial antibiotics | - | 1 (20) | 3 (33) | 4 (20) |
| Necrotizing enterocolitis | 1 (17) | - | - | 1 (5) |
| Persistent pulmonary hypertension developed | - | - | 1 (11) | 1 (5) |
| Spontaneous intestinal perforation | - | 1 (20) | - | 1 (5) |
| **Type of non-trial antibiotic and anti-fungal treatment** | | | | |
| Cefotaxime | - | 1 | 1 | 2 |
| Ceftazidime | - | - | 1 | 1 |
| Meropenem | 1 | 1 | 2 | 4 |
| Meropenem, colistin | - | 1 | - | 1 |
| Meropenem, vancomycin | 1 | - | 1 | 2 |
| Piperacillin/tazobactam, amikacin | 4 | 2 | 4 | 10 |
| Fluconazole | 2 | - | - | 2 |
| Amphotericin B | 1 | - | 1 | 2 |
| **Pathogens isolated from sterile sites** | | | | |
| Candida non-albicans spp | 2 | - | 2 | 4 |
| *Klebsiella pneumoniae* | 1 | 1 | - | 2 |
| *Pseudomonas stutzeri* ^1^ | - | 1 | - | 1 |
| *Serratia liquefaciens* | - | 1 | - | 1 |
| *Enterococcus faecium* | - | - | 1 | 1 |
| *Enterococcus faecalis* | - | - | 1 | 1 |
| *Staphylococcus aureus* | - | - | 1 | 1 |

*Numbers are n (%) or median [min, max];* ^1^ all pathogens were identified from blood cultures, except this one

identified from cerebrospinal fluid.

Supplementary Figure 1: Goodness of fit diagnostics of the final fosfomycin model

(A) Observed fosfomycin concentrations versus individual predicted concentrations (IPRED); (B) observed fosfomycin concentrations versus population predicted concentrations (PRED); (C) conditional weighted residuals (CWRES) versus PRED; (D) CWRES versus time after dose. The blue circles represent observed fosfomycin concentrations, and the solid red lines indicate the line of identity


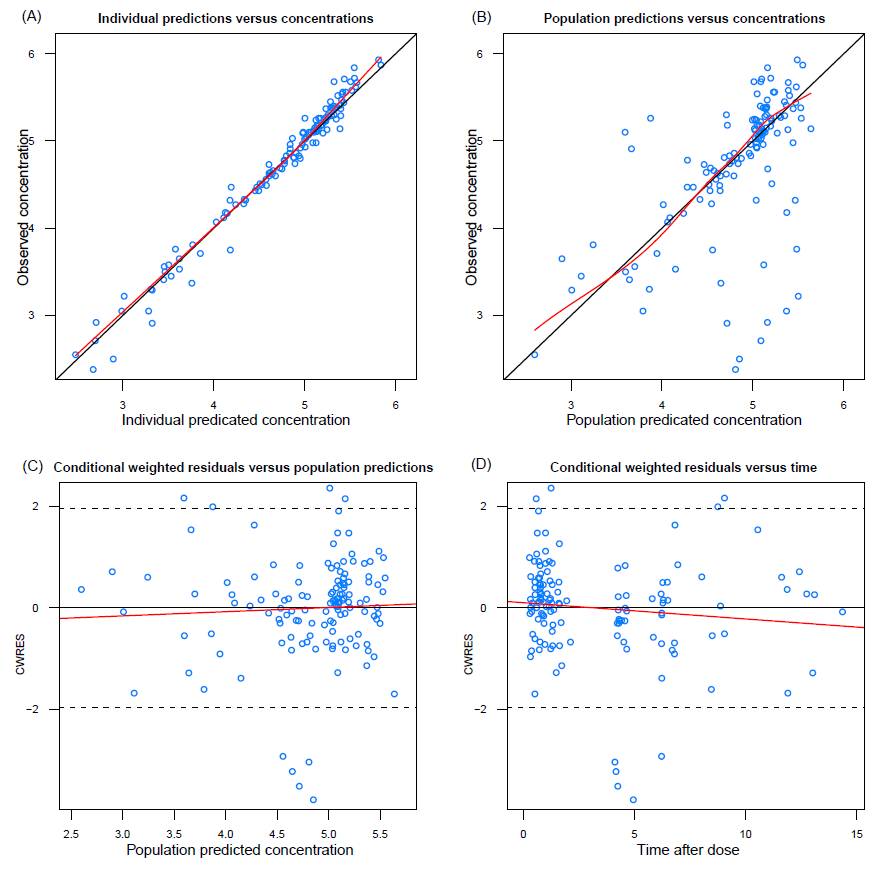


Supplementary Figure 2: Visual predictive check of final fosfomycin model

Open circles represent the observed data. The lower, middle and upper lines are the 5^th^, 50^th^ and 95^th^ percentiles of the observed data. The shaded areas are the 95% confidence intervals of the 5^th^, 50^th^ and 95^th^ percentiles of the simulated data (n=1,000)


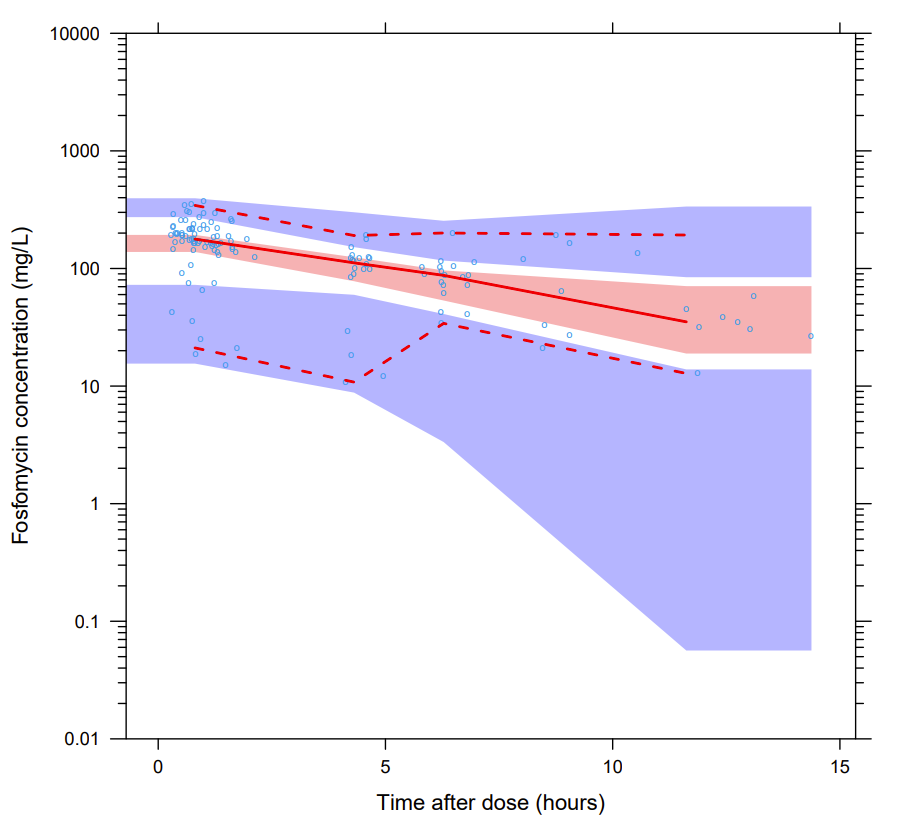


Supplementary Table 5: Population PK parameter estimates from the final fosfomycin PK model

| **Fosfomycin PK parameters** | **Final model** | | **SIR** | |
| --- | --- | --- | --- | --- |
|  | **Estimates** | **RSE (%)** | **Median** | **2.5^th^ - 97.5^th^ percentile** |
| CL (L/hr/70 kg)^a^ | 10.6 | 17.7 | 10.6 | 8.7 - 12.7 |
| θ_WT_ | 0.75 FIX | - | - | - |
| Vc (L/70 kg) ^a^ | 14.8 | 33.2 | 15.0 | 8.2 - 24.6 |
| θ_WT_ | 1.0 FIX | - | - | - |
| Q (L/hr/70 kg) ^a^ | 29.4 | 23.3 | 30.2 | 18.9 - 46.8 |
| Vp(L/70 kg) ^a^ | 22.0 | 17.0 | 22.2 | 17.9 - 25.7 |
| Fraction of CL on the 1^st^ day of life (${}_{M}$) | 0.542 | 20.3 | 0.539 | 0.316 - 0.752 |
| Post-natal maturation rate constant (/day) (${}_{N}$) | 0.361 | 92.0 | 0.367 | 0.186 - 0.708 |
| Inter-individual variability (IIV)  IIV CL, %CV  IIV Vc, %CV  IIV Q, %CV  IIV Vp, %CV | 44.4  230.1  -  - | 14.8  24.5  -  - | 44.7  232.0  -  - | 34.5 - 58.4  149.5 - 375.5  -  - |
| Residual variability | 0.0293 | 27.8 | 0.0299 | 0.0217 - 0.0383 |

*^a^Parameter estimates are scaled to typical patient with body weight of 70 kg. Abbreviations: CL, clearance; Vc, volume of distribution of central compartment; Q, inter-compartment clearance; Vp, volume of distribution of peripheral compartment; RSE%: relative standard error (standard error of estimate / estimate*100); SIR,* *sampling importance resampling*

Final Pharmacokinetic Parameter Equations:

| $CL \left( L/{hr} \right)=\left( 10.6 \right).\left( \frac{{BW}_{i}}{70} \right)^{0.75}.\left( \frac{{PMA}_{i}^{3.4}}{{47.7}^{3.4}+{PMA}_{i}^{3.4}} \right).\left[ 0.542+\left( 1-0.542 \right).\left( {1-e}^{\left( {-PNA}_{i} \right)\left( 0.361 \right)} \right) \right].e^{{}_{i}}$ |
| --- |
|  |
| $Vc \left( L \right)=\left( 14.8 \right).\left( \frac{{BW}_{i}}{70} \right).e^{{}_{i}}$ |
|  |
| $Q \left( L/{hr} \right)=\left( 29.4 \right).\left( \frac{{BW}_{i}}{70} \right)^{0.75}.e^{{}_{i}}$ |
|  |
| $Vp\left( L \right)=\left( 22.0 \right).\left( \frac{{BW}_{i}}{70} \right).e^{{}_{i}}$ |

Supplementary Figure 3: Goodness of fit plots of the final flomoxef model

(A) Observed flomoxef concentrations versus individual predicted concentrations (IPRED); (B) observed flomoxef concentrations versus population predicted concentrations (PRED); (C) conditional weighted residuals (CWRES) versus PRED; (D) CWRES versus time after dose. The blue circles represent observed flomoxef concentrations, and the solid red lines indicate the line of identity


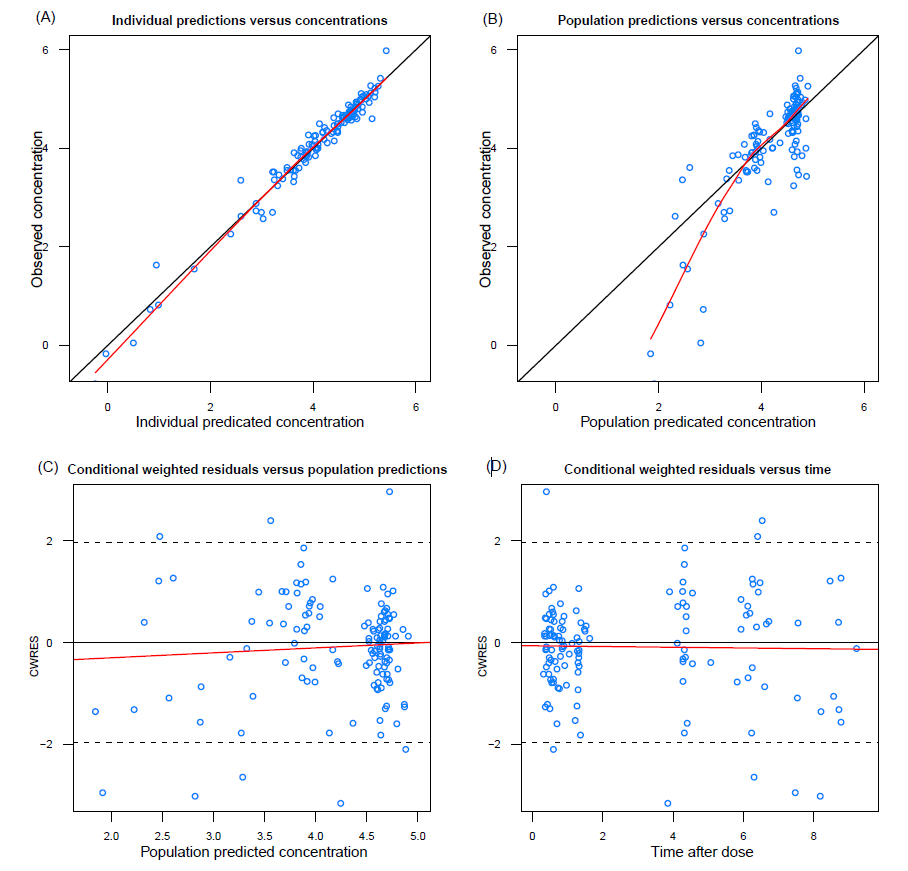


Supplementary Figure 4: Visual predictive checks for the final flomoxef model

Open circles represent the observed data. The lower, middle and upper lines are the 5^th^, 50^th^ and 95^th^ percentiles of the observed data. The shaded areas are the 95% confidence intervals of the 5^th^, 50^th^ and 95^th^ percentiles of the simulated data (n=1,000)


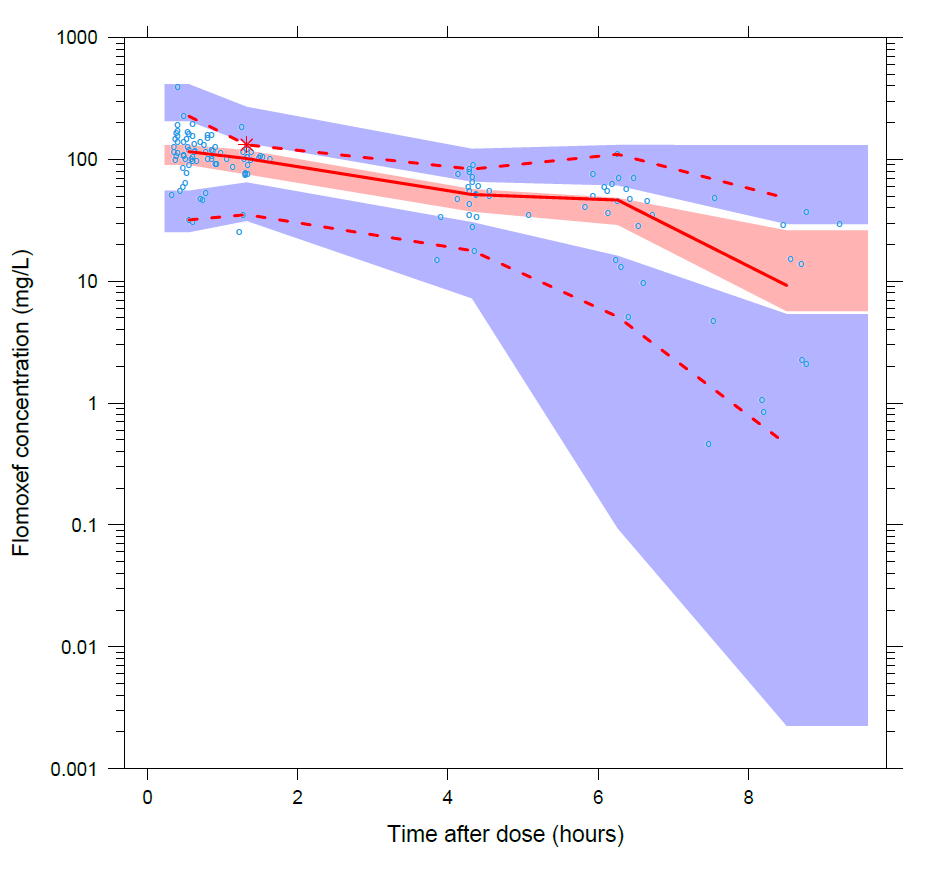


Supplementary Table 6: Population PK parameter estimates from the final flomoxef PK model

| **Flomoxef PK parameters** | **Final model** | | **SIR** | |
| --- | --- | --- | --- | --- |
|  | **Estimates** | **RSE (%)** | **Median** | **2.5^th^ - 97.5^th^ percentile** |
| CL (L/hr/70 kg)^a^ | 12.6 | 5.6 | 12.6 | 11.2 - 14.0 |
| θ_WT_ | 0.75 FIX | - | - | - |
| Vd (L/70 kg)^a^ | 23.9 | 8.4 | 23.8 | 20.3 - 28.1 |
| θ_WT_ | 1.0 FIX | - | - | - |
| Fraction of CL on the 1^st^ day of life (${}_{M}$) | 0.449 *fixed*^b^ | - | - | - |
| Post-natal maturation rate constant (/day) (${}_{N}$) | 0.117 *fixed*^b^ | - | - | - |
| Inter-individual variability (IIV)  IIV CL, %CV  IIV Vd, %CV | 27.8  52.3 | 14.7  18.2 | 27.9  52.6 | 22.4 - 32.1  42.9 - 70.6 |
| Residual variability | 0.0634 | 31.7 | 0.0642 | 0.0475 - 0.0926 |

*^a^Parameter estimates are scaled to typical patient with body weight of 70 kg. ^b^The values of* $q_{M}$ *and* $q_{N}$ *were fixed to reported values from the NeoFos study.(14) Abbreviations: CL, clearance; Vd, volume of distribution; RSE%: relative standard error (standard error of estimate / estimate*100); SIR,* *sampling importance resampling*

Final Pharmacokinetic Parameter Equations:

| $CL \left( L/{hr} \right)=\left( 12.6 \right).\left( \frac{{BW}_{i}}{70} \right)^{0.75}.\left( \frac{{PMA}_{i}^{3.4}}{{47.7}^{3.4}+{PMA}_{i}^{3.4}} \right).\left[ 0.449+\left( 1-0.449 \right).\left( {1-e}^{\left( {-PNA}_{i} \right)\left( 0.117 \right)} \right) \right].e^{{}_{i}}$ |
| --- |
|  |
| $Vd \left( L \right)=\left( 23.9 \right).\left( \frac{{BW}_{i}}{70} \right).e^{{}_{i}}$ |
|  |

Supplementary Figure 5: Box-plot of predicted AUC on Days 1 and Day 5 for (a) fosfomycin and (b) flomoxef


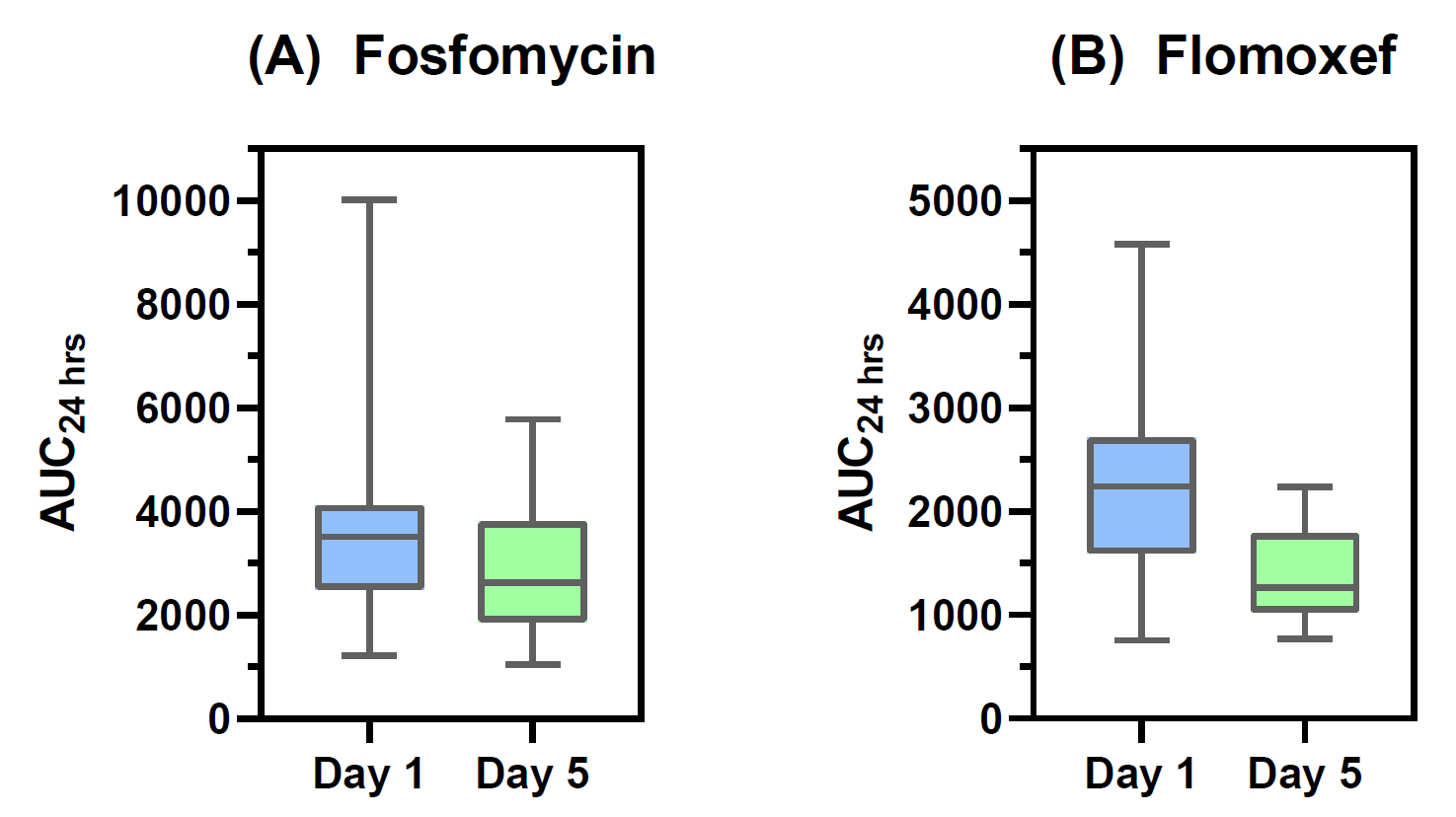


**Supplementary Table 7. Probability of target attainment (PTA) in neonates with different PNA administered fosfomycin every 12 hours achieving target AUC_24 hr_/MIC ratios of 83**

| **MIC (mg/L)** | **%PTA** | | | |
| --- | --- | --- | --- | --- |
|  | **Preterm neonates**  **(GA at birth < 37 weeks)** | | **Term neonates**  **(GA at birth > 37 weeks)** | |
|  | **PNA 1-7 days** | **PNA > 8 days** | **PNA 1-7 days** | **PNA > 8 days** |
| **AUC_24 hr_/MIC > 83** | | | | |
| 1 | 100 | 100 | 100 | 100 |
| 2 | 100 | 100 | 100 | 100 |
| 4 | 100 | 100 | 100 | 100 |
| 8 | 99.91 | 99.73 | 99.95 | 99.72 |
| 16 | 96.11 | 91.54 | 96.58 | 88.68 |
| 32 | 63.40 | 47.78 | 63.22 | 36.72 |
| 64 | 14.40 | 6.48 | 12.32 | 2.89 |

*AUC_24 hr_/MIC, 24-hours area under the concentration-time curve over the minimum inhibitory concentration;* *PTA, probability of target attainment; GA, gestational age; PNA, postnatal age*

**Supplementary Table 8. Probability of target attainment (PTA) in neonates with different PNA administered flomoxef every 8 hours achieving target %fT>MIC of 40.**

| **MIC (mg/L)** | **%PTA** | | | |
| --- | --- | --- | --- | --- |
|  | **Preterm neonates**  **(GA at birth < 37 weeks)** | | **Term neonates**  **(GA at birth > 37 weeks)** | |
|  | **PNA 1-7 days** | **PNA > 8 days** | **PNA 1-7 days** | **PNA > 8 days** |
| 0.06 | 100 | 99.99 | 100 | 99.94 |
| 0.12 | 100 | 99.99 | 100 | 99.86 |
| 0.25 | 100 | 99.98 | 100 | 99.77 |
| 0.5 | 99.99 | 99.95 | 99.99 | 99.61 |
| 1 | 99.99 | 99.88 | 99.98 | 99.28 |
| 2 | 99.99 | 99.72 | 99.96 | 98.58 |
| 4 | 99.99 | 99.31 | 99.86 | 96.95 |
| 8 | 99.94 | 98.18 | 99.52 | 92.35 |
| 16 | 99.55 | 93.53 | 97.54 | 78.02 |
| 32 | 94.93 | 71.74 | 78.55 | 32.87 |
| 64 | 50.67 | 17.95 | 12.84 | 1.09 |

*PTA, probability of target attainment; GA, gestational age; PNA, postnatal age*

Supplementary Table 9: Clinical features of the seven neonatal deaths

| **Death No** | **Cohort** | **GA (weeks)** | **Birth weight (grams)** | **Sex** | **Cause of Death** | **Died on trial antibiotics** | **Age at death (days)** |
| --- | --- | --- | --- | --- | --- | --- | --- |
| 1 | Cohort 1 | 32 | 1320 | F | Necrotizing enterocolitis | N | 20 |
| 2 | Cohort 1 | 31 | 1220 | F | Culture negative sepsis | Y | 17 |
| 3 | Cohort 1 | 34 | 1500 | M | Culture positive sepsis ^1^ | N | 17 |
| 4 | Cohort 2 | 39 | 2900 | M | Culture negative sepsis | Y | 4 |
| 5 | Cohort 2 | 34 | 1160 | M | Culture negative sepsis | N | 5 |
| 6 | Cohort 2 | 30 | 1120 | F | Culture positive sepsis ^2^ | N | 9 |
| 7 | Cohort 3 | 31 | 1320 | F | Persistent pulmonary hypertension | N | 3 |

*GA: gestational age; F=female; M=male; N=No; Y=Yes, 1= Enterobacter spp. and Klebsiella spp. and Candida non-albicans; 2 = Serratia liquefaciens*

Supplementary Table 10: Adverse events related to trial antibiotics

| **Neonate No** | **Cohort** | **Event** | **Maximum grade** | **Related to fosfomycin** | **Related to flomoxef** | **Related to amikacin** |
| --- | --- | --- | --- | --- | --- | --- |
| 1 | Fosfomycin/amikacin | Neutropenia | Grade 1 | yes |  |  |
| 2 | Fosfomycin/amikacin | Blood urea increased | Grade 2 |  |  | yes |
|  | Fosfomycin/amikacin | Hypernatremia | Grade 2 | yes |  |  |
| 3 | Flomoxef/amikacin | Administration site complication | Grade 1 |  | yes |  |
| 4 | Flomoxef/amikacin | Sinus bradycardia | Grade 1 |  | yes | yes |
| 5 | Flomoxef/amikacin | Blood creatinine increased | Grade 1 |  |  | yes |
|  | Flomoxef/amikacin | Blood urea increased | Grade 2 |  | yes | yes |
|  | Flomoxef/amikacin | Hypernatremia | Grade 2 |  | yes |  |
| 6 | Flomoxef/amikacin | Aspartate aminotransferase increased | Grade 1 |  | yes |  |
|  | Flomoxef/amikacin | Blood creatinine increased | Grade 2 |  | yes | yes |
| 7 | Flomoxef/amikacin | Blood creatinine increased | Grade 2 |  | yes | yes |
| 8 | Fosfomycin/flomoxef | Hepatic function abnormal | Grade 2 | yes | yes |  |
| 9 | Fosfomycin/flomoxef | Hypokalemia | Grade 3 | yes |  |  |
| 10 | Fosfomycin/flomoxef | Hepatic function abnormal | Grade 3 | yes | yes |  |
| 11 | Fosfomycin/flomoxef | Hypokalemia | Grade 2 | yes |  |  |
| 12 | Fosfomycin/flomoxef | Hepatic function abnormal | Grade 2 | yes | yes |  |
| 13 | Fosfomycin/flomoxef | Acute kidney injury | Grade 2 | yes | yes |  |
|  | Fosfomycin/flomoxef | Hepatic function abnormal | Grade 2 | yes | yes |  |
|  | Fosfomycin/flomoxef | Hypernatremia | Grade 1 | yes |  |  |

Supplementary Table 11: NeoSep Severity Score for predicting 28-day mortality based on clinical information at the start of a sepsis episode

| **Factor (clinical signs in the 24h preceding start of clinical sepsis episode)** | **Score value if present** |
| --- | --- |
| Time in hospital: ≤ 10 days | 1 |
| Gestational age: <37 weeks | 1 |
| Birth Weight:   - >2 kg - 1-2 kg - <1 kg | 0 1 2 |
| Congenital anomalies | 2 |
| Temperature   - <35.5°C - 35.5 to 37.9 °C - 38 – 38.9 °C - ≥ 39 °C | 1  0  1  2 |
| Maximum respiratory support:   - None - Oxygen supplementation - CPAP, BiPAP, HFNC - Invasive ventilation | 0 2 3  3 |
| Abdominal distension | 1 |
| Difficulty in feeding | 1 |
| Evidence of shock including cold peripheries | 1 |
| Lethargy / no or reduced movement:   - Lethargy only - No movement or movement only on stimulation +/- lethargy | 1  2 |

*Note: CPAP = continuous positive airway pressure, BiPAP = Bilevel Positive Airway Pressure, HFNC = high flow nasal cannula. The score* *was adapted from WHO possible serious bacterial infection (pSBI) criteria for hospitalised neonates with sepsis and based on the data generated from the NeoOBS study (Russell NJ, et al. PLoS Med. 2023;20(6):e1004179)*

Supplementary Table 12: Trial Assessment Schedule (Part 1)

| **Visit type** | **Screening** | **Enrolment** | **Follow-up Treatment & Monitoring** | | | | | **TOC** | **StFU*** |
| --- | --- | --- | --- | --- | --- | --- | --- | --- | --- |
| **Timing (window)** | **Day 0** | **Day 1** | **Daily while on IV antibiotics** | **Day 3**  **(±1 day)** | **Day 5 (±1 day)** | **Day 7  (±2 day)** | **EOT ^8^**  **(if not Day 7 or 14)** | **14**  **(± 4 days)** | **28**  **(± 5 days)** |
| Informed assent/consent | x^1^ |  |  |  |  |  |  |  |  |
| Verification of eligibility | x | X |  |  |  |  |  |  |  |
| Enrolment to Part 1 |  | x^2^ |  |  |  |  |  |  |  |
| Medical history | x | X |  |  |  |  |  |  |  |
| Clinical review | x | X | x | x | x | x | x | x | x |
| C-reactive Protein | x^3^ |  |  |  | x | x |  |  |  |
| Full Blood Count (FBC) | x^3^ |  |  |  | x | x^6^ | x^6^ | x^6^ | x^6^ |
| Urea & Electrolytes (U&Es) | x^3^ |  |  |  | x | x^6^ | x^6^ | x^6^ | x^6^ |
| Liver function test (LFT) | x^3^ |  |  |  | x | x^6^ | x^6^ | x^6^ | x^6^ |
| Creatinine | x^3^ |  |  |  | x | x^6^ | x^6^ | x^6^ | x^6^ |
| Blood culture | x^4^ |  |  | x^5^ |  |  |  |  |  |
| Administration of antibiotics |  | X | X | x | x | x | x |  |  |
| Pharmacokinetic sample^7^ |  | X |  |  | x |  |  |  |  |
| Adverse event assessment |  | X | X | x | x | x | x | x | x |
| Concomitant medication |  | X | X | x | x | x | x | x | x |

EOT= end of treatment, TOC = test of cure, StFU = short term follow-up visit. Last FU visit for Part 1 participants will be on Day 28.

* by telephone / if clinically indicated, then hospital visit.

^1^ Written informed consent to be obtained from parent/guardian.

^2^ Treatment allocation in Part 1 and treatment initiation may be on the same day as the screening visit.

^3^ Laboratory results required within 48h before enrolment, but test can be done either at screening or randomisation or values from blood taken pre-screening.

FBC: Red blood count (RBC), white blood count (WBC) and differential, platelets. U&Es: including blood urea nitrate (BUN), sodium, potassium. LFTs: ALT, AST.

^4^ Blood must be taken for culture within 48h before enrolment, but may precede screening visit by up to 48 hours if already taken for clinical management.

^5^ Repeat blood culture only if neonate switches treatment (at the time of switch) due to clinical deterioration or lack of response. Blood for culture should be taken before switch of antibiotics except in exceptional circumstances outside the responsible clinician’s control.

^6^ Repeat blood tests only if abnormal at previous visit or baby’s condition not stable.

^7^ Pharmacokinetic samples for Part 1. PK sample from CSF may also be collected if lumbar puncture is clinically indicated and baby receiving fosfomycin.

^8^ Planned duration of treatment at enrolment for blood culture-negative sepsis is to Day 7±2 days, for blood culture-positive sepsis is to Day 10 [-3,+4 days] if there is no switch to second-line. If antibiotics are switched to second-line, the total planned duration of antibiotic treatment including first and second line treatment is 14 ±7 days depending on the baby’s condition.
